# Description and genomic characterization of *Streptococcus symci* sp. nov., isolated from a child’s oropharynx

**DOI:** 10.1007/s10482-020-01505-3

**Published:** 2021-01-02

**Authors:** He Qi, Defeng Liu, Yang Zou, Nan Wang, Han Tian, Chunling Xiao

**Affiliations:** 1 Liaoning University of Traditional Chinese Medicine, Shenyang, People’s Republic of China; 2 Key Lab of Environmental Pollution and Microecology of Liaoning Province, Shenyang Medical College, No. 146, Huanghe North Street, Shenyang, Liao Ning People’s Republic of China; 3 Department of Medical technology, Medical Science Institute of Liaoning, Shenyang, People’s Republic of China

**Keywords:** Culturomics, Human oral microbiota, *Streptococcus symci*, New species, Taxono-genomics

## Abstract

Using the culturomics approach, we isolated a new *Streptococcus* species, strain C17^T^, from the oropharynx mucosa sample of a healthy 5-year-old child living in Shenyang, China. We studied the phenotypic, phylogenetic, and genomic characteristics of strain C17^T^, which was identified as a Gram-positive, coccus-shaped, non-motile, aerobic, catalase-negative bacteria. Its growth temperatures ranged from 20 to 42 °C, with optimal growth at 37 °C. Acid production could be inhibited by two sugars, trehalose and raffinose. In C17^T^, the reactions for enzyme lipase (C14) were confirmed to be negative, whereas those for alkaline phosphatase, α-glucosidase, and hippuric acid hydrolysis were positive. The C17^T^ genome contained 2,189,419 base pairs (bp), with an average G+C content of 39.95%, encoding 2092 genes in total. The 16S ribosomal RNA sequence showed 99.8% similarity with the newly identified *Streptococcus pseudopneumoniae* ATCC BAA-960^T^. The main fatty acid components in C17^T^ were C16:0, C18:1 w7c, C18:0, and C18:1 w9c, all of which can be found in other species of the *Streptococcus* genus. Strain C17^T^ showed high susceptibility to clindamycin, linezolid, vancomycin, chloramphenicol, and cefepime, and moderate susceptibility to erythromycin. The obtained dDDH value between strain C17^T^ and the closest species was 52.9%. In addition, the whole genome sequence of strain C17^T^ had an 82.21–93.40% average nucleotide identity (ANI) with those strains of closely related *Streptococcus* species, indicating that the strain C17^T^ was unique among all Streptococcus species. Based on these characteristics, we determine that C17^T^ is a novel species, named *Streptococcus symci* sp. nov. (= GDMCC 1.1633 = JCM 33582).

## Introduction

As an important part of the salivary microbiome, *Streptococcus* comprises 107 officially identified species (https://lpsn.dsmz.de/genus/streptococcus), which have been divided into six different groups according to 16S ribosomal RNA (rRNA) sequence results (*anginosus*, *bovis*, *mitis*, *mutans*, *pyogenic*, and *salivarius*) (http://www.bacterio.net/) (Kawamura et al. 1995). Many *Streptococcus mitis* strains are highly virulent, which result in various pathologies, such as meningitis, endocarditis, and pneumonia, by invading the normal microbial community of low pathogenic commensal species (Ricaboni et al. 2017). In a previous study, four antagonistic *Streptococcus* strains isolated from oropharyngeal microbiota were found to have bacteriostatic effects on pathogens and were involved in pharyngeal microbiome homeostasis (Li et al. 2019). The pathogenic and commensal species isolated from the upper respiratory tract of healthy people exhibited similar morphology on culture medium and were distinguished correctly and rapidly, especially species sharing a 16S rRNA sequence identity greater than 98.7%. This is of great significance for screening probiotics and monitoring disease epidemiology for clinical applications (Arbique et al. 2004). Therefore, it is necessary to use more accurate and rapid methods for their identification. Generally, the methods that are typically used include high-throughput screening together with matrix-assisted laser desorption/ionization-time of flight or 16S rRNA sequencing to identify isolated colonies in order to study the microbial community (Bittar et al. 2014). Several different housekeeping genes have been amplified for analysis, including *sodA* (Poyart et al. 1998), *rpoB* (Tapp et al. 2003), and *groEL* (Glazunova et al. 2009), which are recognized as the classification criteria for determining novel *Streptococcus* species (Okamoto et al. 2015; Vela et al. 2015; Vela et al. 2016). DNA-DNA hybridization (DDH) is a key criterion for the identification of new species (Auch et al. 2010). Average nucleotide identity (ANI) exhibits a strong correlation with DNA-DNA hybridization (DDH) values, with an ANI value ≥ 95% corresponding to the traditional 70% DDH threshold.

In this study, we analyzed the characteristics of a novel species by using a series of cultivation and genetic manipulations (Ramasamy et al. 2014) including phenotype identification, housekeeping gene sequencing, phylogenetic analysis, genome sequencing and annotation, fatty acid methylester analysis, and the antibiotic susceptibility test. This novel *Streptococcus* species was verified and named *Streptococcus symci* C17^T^ (= GDMCC 1.1633 = JCM 33582).

## Materials and methods

### Sample collection and strain isolation

In this study, pharyngeal swabs were used to collect bacterial samples. Strain C17^T^ was isolated from the oropharynx mucosa sample of a healthy 5-year-old child living in Shenyang, China in December 2015. After sample collection, the pharyngeal swab was immediately preserved in a 4 °C transport medium, sent to the laboratory, and stored at − 80 °C. The bacterial samples obtained were dissolved in 1 mL brain heart infusion (BHI) broth (bioMérieux, Craponne, France) and subsequently diluted to 1 L for further experiments. Approximately 200 μL of the sample mixture was spread onto Columbia agar (bioMérieux) supplemented with 5% (vv) defibrinated sheep blood (Solarbio, Beijing, China) and incubated at 37 °C aerobically in 5% CO_2_ for 24 h. Circular single colonies surrounded by a zone of α-hemolysis were picked from the plates using an inoculation needle, re-streaked on Columbia blood agar, and incubated at 37 °C for another 24 h. Separate colonies were chosen from the plate and cultured in liquid medium until subsequent use.

### Strain identification and gene sequencing of 16S rRNA, *groEL*, *rpoB*, and *sodA*

Genomic DNA was isolated from the bacterial colonies using the Wizard Genomic DNA Purification Kit (Promega, Madison, WI, USA), and 16S rRNA gene sequencing was conducted using the protocol described by Delgado et al. (2006) and Jin et al. (2013). The universal eubacterial primers 27F/1492R (27F:5′ - agagtttgatcmtggctcag -3′ and 1492R:5′ - ggytaccttgttacgactt -3′) were applied during PCR analysis using the Gene Amp PCR System 3730 Thermal Cycler (ABI, Vernon, CA, USA), as previously described (Drancourt et al. 2000). A nucleotide basic local alignment search tool (BLASTn) analysis (Altschul et al. 1990) was performed and aligned within the national center for biotechnology information (NCBI) database. Gene alignment results indicated that the strain belonged to the *Streptococcus* genus. The *groEL*, *rpoB*, and *sodA* genes of strain C17^T^ were amplified using the primer pairs streptogroELd/streptogroELr, 1730_F/3700_R, and d1/d2, respectively, as previously described (Drancourt et al. 2004; Glazunova et al. 2009; Poyart et al. 2002). Subsequently, BLAST analysis of these three genes was performed using default NCBI parameters.

### Phylogenetic analysis

The genome sequences of the *Streptococcus* genus were obtained from the database list of prokaryotic names with standing in nomenclature (http://www.bacterio.net/streptococcus.html). The taxon of *Streptococcus* is based on Bergey’s Manual of Systematics of Archaea and Bacteria. The 16S rRNA genes of the new *Streptococcus* isolates were sequenced, aligned with those of other species of *Streptococcus* strains and related taxa. Phylogenetic trees were constructed and genetic distances were calculated using NCBI analysis (https://www.ncbi.nlm.nih.gov/nucleotide/), which was used for the sequence download of phylogenetically closest species. The sequences of *groEL, rpoB*, and *sodA* from the closest species with standing in nomenclature were directly downloaded from the NCBI after BLASTn analysis. The phylogenetic tree in this study was reconstructed with concatenated *groEL*, *soda*, and *rpoB* sequences of strain C17^T^ and other closely related species. Alignment was performed using MEGA X software (Tamura et al. 2013; Kumar et al. 2018). The neighbor-joining method was applied for phylogenetic inference generation. Bootstrap analysis (1000 replications) was performed to assess the reliability of the nodes.

### Morphologic observation and optimal growth conditions

After 24 h of incubation, the bacterial cells were Gram-stained and observed using a Leica DM 500 photonic microscope (Leica Microsystems, Nanterre Cedex, France) with a 100 × oil immersion lens. Cell morphology was determined using a scanning electron microscope (Hitachi, Tokyo, Japan) set to the following conditions: accelerating voltage 30,000 V, magnification 7000, working distance 6700 μm, and emission current 112,000 nA. Cell motility was evaluated on soft agar plates (Xu et al. 2013). To determine the optimal culture conditions, several culture conditions were tested for strain C17^T^. Culture assays were performed on Columbia agar supplemented with 5% defibrinated sheep blood (bioMerieux) at temperatures ranging from 4 to 45 °C (4 °C, 15 °C, 20 °C, 22 °C, 25 °C, 30 °C, 35 °C, 37 °C, 42 °C and 45 °C). The salt tolerance of strain C17^T^ was tested at various NaCl concentrations (1.5%, 2.0%, 2.5%, 3.0%, 3.5%, 4.5% and 6.5%). The oxygen demand was tested under aerobic, anaerobic, and microaerophilic (GENbag; BioMerieux) conditions. Different pH values (from 4.0 to 10.0) were also tested. Hemolytic activity was observed on Columbia blood agar plates. Catalase assays (bioMerieux) were performed following standard protocols. The oxidase reaction was assessed using the Becton Dickinson oxidase reagent (Becton Dickinson, Franklin Lakes, NJ, USA).

### Biochemical and fatty acid methylester analysis and antibiotic susceptibility test

#### Biochemical analysis

The identification of API 50CH, API20 NE, and API ZYM (bioMerieux) was used to distinguish *Bacilli*, *Enterococcus*, and adjacent *Streptococcus* strains with a positive enzyme test, and the experiments were carried out according to standard instructions.

#### Fatty acid analysis

Each tube of samples was prepared using approximately 30 mg of bacterial biomass harvested from several Columbia agar plates supplemented with 5% sheep blood. Cellular fatty acids were then extracted, modified, and analyzed according to the standard protocol, using gas chromatography (Agilent 7890; Agilent Technologies, Santa Clara, CA, USA) coupled with the Sherlock Microbial Identification System Version 6.3 (MIDI Inc., Newark, DE, USA).

#### Antibiotic susceptibility testing

The antibiotic susceptibility of strain C17^T^ was tested on antibiotic-sensitive paper (OXOID) using disk diffusion assays following the Clinical Laboratory Standards Institute 2018 recommendations. The antibiotics used in this study were as follows: clindamycin, 2 μg/mL; linezolid, 30 μg/mL; chloramphenicol, 30 μg/mL; erythromycin, 15 μg/mL; cefepime, 30 μg/mL; vancomycin, 30 μg/mL; ampicillin, 10 μg/mL; ceftriaxone, 30 μg/mL; and cefotaxime, 30 μg/mL.

### Genomic DNA extraction, genome sequencing, and assembly

Genomic DNA was extracted using the EZ1 DNA Tissue Kit (Qiagen, Hilden, Germany) according to the standard protocol. The DNA obtained was validated using gel electrophoresis and quantified using a Qubit® 2.0 Fluorometer (Thermo Fisher Scientific, Waltham, MA, USA). Approximately 1 μg of total DNA from each sample was used for sequencing. Libraries for sequencing were constructed using the NEBNext® Ultra™ DNA Library Prep Kit for Illumina (New England Biolabs, Ipswich, MA, USA) following standard recommendations, and index codes were included to attribute sequences to each sample. Each DNA sample was fragmented by sonication to an average size of 350 base pairs (bp). The fragments obtained were end-polished and A-tailed and then ligated with the adapter for further PCR amplification. Illumina PCR adapter reads and low-quality reads were discarded after the quality control step using their compilation pipeline. All paired-end reads with good quality were assembled using the SOAP denovo online software (Li et al. 2008; Li et al. 2010) (http://soap.genomics.org.cn/soapdenovo.html) into different DNA contigs that were handled by the next step for gap closing. PCR products were purified (AMPure XP PCR purification system; Beckman Coulter, Brea, CA, USA), and libraries for size distribution were analyzed with the Agilent 2100 Bioanalyzer and quantified using quantitative PCR. The genome of C17^T^ was sequenced using the Illumina NovaSeq PE150 facility (Illumina Inc., San Diego, CA, USA) at the Beijing Novogene Bioinformatics Technology Co., Ltd. (Beijing, China). Raw data were further processed in four steps: discarding the reads of low-quality (≤ Q20) bases and N-base to reach a certain proportion of reads (default is 10%); discarding the reads whose overlap with adapter exceeded a certain threshold value (default value is 15 bp) and mismatch number < 3; and removing adapter and duplication contamination. Finally, 100 × coverage of reads was obtained with clean paired-end read data. The genome size was estimated using k-mer statistical analysis before assembly. Data were assembled with SOAP denovo (version 2.04) and validated with SPAdes (Bankevich et al. 2012) and ABySS (Simpson et al. 2009) assemblers. Finally, the software CISA (Lin and Liao. 2013) was used for integration. Gap close (version 1.12) software was used to optimize and mend the initial assembly results to obtain the final assembly results. Fragments below 500 bp were filtered out.

### Genome annotation and analysis

For the final assembled results of each sample to be ≥ 500 bp, open reading frames were annotated using Prodigal with standard settings (http://prodigal.ornl. gov/) (Hyatt et al. 2010). The GeneMarkS program (Besemer et al. 2001) (http://topaz.gatech.edu/genemark/) was used to predict the coding region of the newly sequenced genome. Transfer RNA (tRNA), rRNA, and small nuclear RNA genes were analyzed using tRNAscan-SE (Lowe and Eddy 1997), rRNAmmer (Lagesen et al. 2007), and Pfam (Gardner et al. 2009; Nawrocki et al. 2009) databases. The interspersed repetitive sequences were analyzed using Repeat Masker (Saha et al. 2008) (http://www.repeatmasker.org/). Tandem repeats were analyzed using a tandem repeats finder (Benson 1999). The Island Path-DIOMB program (Hsiao et al. 2003) and transposon PSI were used to predict the genomic islands and transposons based on the homologous BLAST method. Prophage prediction was carried out by PHAST9 (Zhou et al. 2011) (http://phast.wishartlab.com/), and clustered regularly interspaced short palindromic repeat sequences (CRISPRs) were identified using CRISPR Finder (Grissa et al. 2007). The basic steps of the annotation function are listed below: The predicted protein sequence was compared with each functional database using diamond (e-value ≤ 1e-5). To filter the comparison results, the results with the highest scores (default identity ≥ 40%, coverage ≥ 40%) were selected for annotation. The bacterial proteome was predicted using the gene prediction program GeneMarkS (version 4.28) together with clusters of orthologous groups (COGs) database. The Pfam (El-Gebali et al. 2019) database was used to analyze protein function by identification of PFAM-A and PFAM-B domains using the hhmscan tool. The secreted proteins were predicted using the Signal P database (Petersen et al. 2011), and the prediction of Type I–VII proteins secreted by the pathogenic bacteria was based on the Effective eT3 software (Eichinger et al. 2016). Meanwhile, the secondary metabolism gene clusters were analyzed using antiSMASH (Medema et al. 2011). To further confirm the novelty of strain C17^T^, the genome-to-genome distance calculator 2.1 (GGDC) was applied to calculate the digital DDH (dDDH), which was estimated with confidence intervals under the recommended settings (Formula 2, http://ggdc.dsmz.de/distcalc2.php).We also measured the overall similarity among compared genomes by using Orthologous Average Nucleotide Identity Tool ( Lee et al. 2015).

## Results

### Phylogenetic analysis

**Fig. 1:**
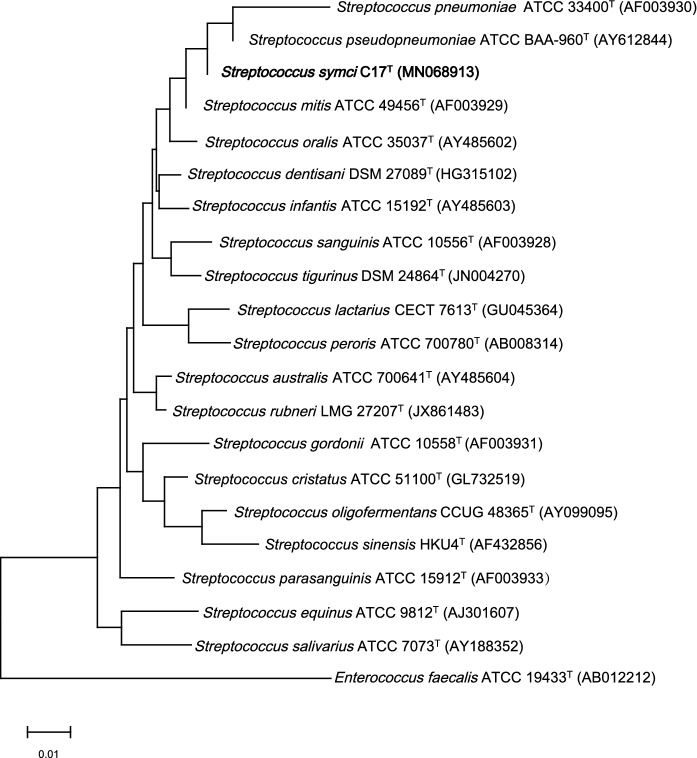
The phylogenetic tree shows the distance between *S. symci* strain C17^T^ and other *Streptococcus* strains. The Gen Bank accession number of each strain is displayed in brackets. Sequences were aligned using ClustalW with standard parameters. Phylogenetic inferences were generated using MEGAX software following the neighbor-joining method with 1000 bootstrap replicates and rooted using *E. faecalis* JCM (AB012212) as the out group. The scale bar represents 1% nucleotide sequence divergence

**Fig. 2:**
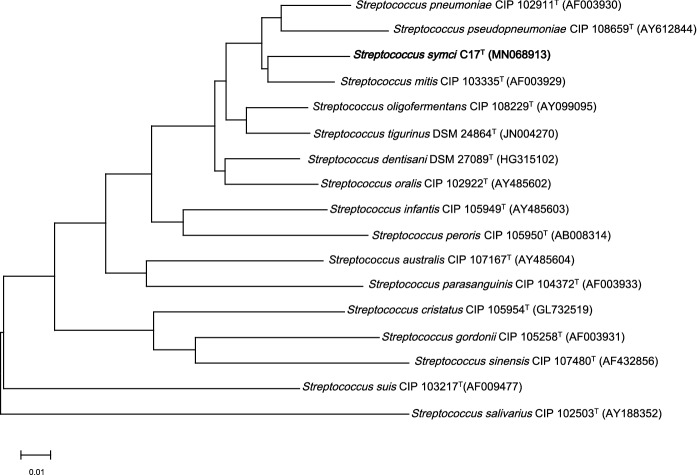
*groEL*, *rpoB*, and *sodA-*based phylogenetic tree showing the position of *S. symci* strain C17^T^ relative to other *Streptococcus* strains. The Gen Bank accession numbers of the strains are shown in brackets. Sequences were aligned using ClustalW with default parameters. Phylogenetic inferences were obtained using MEGAX software following the neighbor-joining method with 1000 bootstrap replicates. The scale bar represents 1% nucleotide sequence divergence

A comparative analysis of the 16S rRNA of strain C17^T^ showed a sequence identity of 99.8% with *S. pseudopneumoniae* strain ATCC BAA-960^T^ (GenBank Accession No. AY612844), 99.6% with *S. pneumoniae* NCTC 7465^T^, and 99.4% with *S. mitis* ATCC 494565^T^, which were the phylogenetically closest species with standing in nomenclature (Fig. 1). The concatenated comparison of sequenced *gro EL*, *rpoB,* and *sodA* indicated that strain C17^T^ and *S. mitis* were in the same branch of the evolutionary tree and had the most recent evolutionary relationship of all the species of *Streptococcus*. This result also revealed that the taxon represented by strain C17^T^ was readily distinguished from its nearest neighbors *S. pseudopneumoniae* strain ATCC BAA-960^T^ and *S. pneumoniae* NCTC 7465^T^ (Fig. 2). The 16S rRNA sequence of strain C17^T^ was deposited in the GenBank with the accession number MN068913.1.

### Phenotypic characteristics and biochemical features

**Fig. 3:**
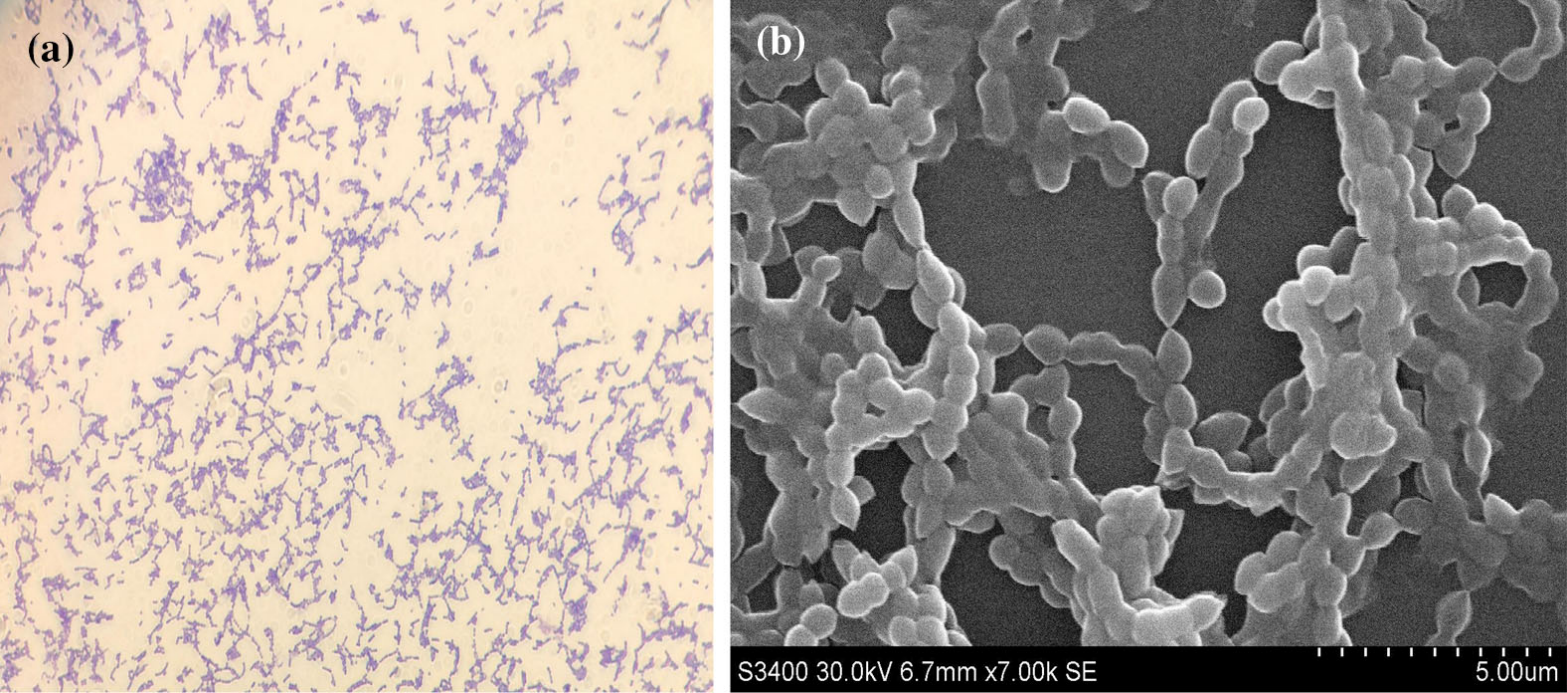
Phenotypic features of *S. symci* C17^T^ **a** Gram-staining of *S. symci* C17^T^. **b** Scanning electron microscopy image of *S. symci* C17^T^ using S-3400N (Hitachi Company) at an operating voltage of 30 keV. Scale bar = 5 μm

Grass-green, α-hemolytic colonies of strain C17^T^ were observed on 5% sheep’s blood-enriched Columbia agar (bioMérieux) after 24 h of incubation under aerobic conditions. Cells were confirmed to be Gram-positive using classical staining (Fig. 3a) and cells with a mean diameter of 5 μm (range, 4–8 μm) and non-spore-forming rods (Fig. 3b) were observed using scanning electron microscopy. The motility assay on soft agar plates revealed the cells were non-motile. C17^T^ displayed a wide range of pH adaptability after a growth test at different pH values (4.0, 4.5, 5.0, 5.5, 6.0, 6.5, 7.0, 7.5, 8.0, 8.5, 9.0, 9.5 and 10.0). To determine salt-tolerance ability, the growth of C17^T^ was observed in up to 2.5% NaCl. The growth of strain C17^T^ was also observed from 20 to 42 °C under anaerobic, microaerophilic, and aerobic conditions. However, no growth was observed at 4 °C, 15 °C, or 45 °C, and the optimal growth was found at 37 °C under aerobic conditions. Catalase and oxidase activity tests were negative. The identification and general characteristics of strain C17^T^ are summarized in Table 1.

**Table 1:** Classification and general features of strain *Streptococcus symci* C17^T^

Property	Term
Classification	Domain *Bacteria* Phylum *Firmicutes* Class *Bacilli* Family *Streptococcaceae* Genus *Streptococcus* Species *Streptococcus symci* Type strain C17^T^
Gram stain	Positive
α-Hemolytic	Positive
Cell shape	Cocci
Motility	Non-motile
Sporulation	Non-spore forming
Oxygen requirement	Aerobe
Temperature range	22–42 °C
Optimum temperature	37 °C
Salt tolerance	< 2.5%
Catalase	Negative
Oxidase tested	Negative
Biotic relationship	Free-living
Origin	Respiratory tract of a healthy child

Strain C17^T^ could be easily distinguished from the nearest phylogenetic neighbors by its specific features, and the biochemical profile of this novel species could also be differentiated from those of closely related species (Table 2), including 16S rRNA gene similarity, lack of acid production from trehalose and raffinose, negative reactions for lipase (C14), and positive reactions for alkaline phosphatase, α-glucosidase, and hippuric acid hydrolysis. Using the API® 20A strip (bioMérieux), positive reactions were only observed for hippuric acid hydrolysis, leucyl-aminopeptidase, and D-lactose fermentation. Negative reactions were observed for the following tests: acid production from starch, esculin hydrolysis, glycogen hydrolysis, pyrrolidinyl arylamidase, Voges-Proskauer reaction, α-galactosidase, β-galactosidase, β-glucuronidase, arginine hydrolase, and fermentation of D-ribose, L-arabinose, D-mannitol, D-raffinose, D-sorbitol, D-trehalose, L-arabinose, and inulin.

**Table 2:** Biochemical characteristics used to differentiate *Streptococcus symci* C17^T^ from the nearest neighbor species of *Streptococcus*

	1	2	3	4	5	6	7
API ZYM							
Alkaline Phosphatase	+	−	−	−	−	+	+
β-Galactosidase	V	−	+	+	+	−	+
α-Glucosidase	+	NA	−	−	−	−	−
β-Glucosidase	+	−	v	−	−	+	−
Lipase (C14)	−	NA	v	+	+	+	v
API 20 Strep							
Voges–Proskauer test	−	−	−	−	−	−	−
Arginine	−	v	−	−	−	−	+
Esculin	−	−	−	V	−	−	−
Hippuric acid	+	−	−	−	−	−	−
Pyrrolidinyl arylamidase	−	NA	+	−	−	−	−
a-Galactosidase	−	+	−	−	−	+	−
Ribose	−	v	v	−	−	−	−
Mannose	−	+	−	−	−	−	−
D-raffinose	−	v	v	−	+	+	−
Starch	−	v	−	+	−	−	−
API 50CH							
D-galactose	+	+	NA	+	+	+	+
D-glucose	+	+	NA	+	+	+	+
D-fructose	+	+	NA	+	+	+	+
Amygdalin	−	−	NA	−	+	−	−
D-mannitol	−	v	−	−	−	−	−
D-sorbitol	−	−	−	−	−	−	−
Melibiose	−	v	−	v	−	−	−
D-tagatose	−	−	−	−	−	−	−
D-lactose	+	+	+	+	+	+	+
D-sucrose	+	+	V	+	+	+	+
D-trehalose	−	V	−	+	+	−	−

Strains: 1, *S. symci* C17^T^; 2, *S.mitis* ATCC 49456^T^; 3, *S. pseudopneumoniae* ATCC BAA-960^T^; 4, *S. oralis* ATCC 35037^T^; 5, *S. infantis* ATCC 15192^T^; 6, *S. dentisani* DSM 27089^T^; 7, *S. australis* ATCC 700641^T^. +, positive reaction; −, negative reaction; V, variable; NA, data not available

Using the API® ZYM strip (bioMérieux), positive reactions were observed as follows: alkaline phosphatase, esterase lipase (C8), trypsin, α-fucosidase, α-glucosidase, α-mannosidase, β-galactosidase, β-glucuronidase, β-glucosidase, N-acetyl-β-glucosaminidase, and cystine arylamidase. Negative reactions were observed as follows: acid phosphatase, arylamidase, esterase (C4), leucine arylamidase, lipase (C14), valine, α-chymotrypsin, α-galactosidase, and naphthol-AS-BI-phosphohydrolase. Using the API® 50CH strip (bioMérieux), positive reactions were observed as follows: D-galactose, D-fructose, D-glucose, D-lactose, D-maltose, D-mannose, D-Sucrose, and N-acetylglucosamine. Negative reactions were observed as follows: D-adonitol, D-arabinose, D-arabitol, D-cellobiose, D-fucose, D-lyxose, D-mannitol, D-melibiose, D-melezitose, D-raffinose, D-ribose, D-saccharose, D-sorbitol, D-tagatose, D-trehalose, D-turanose, D-xylose, methyl-α-D-glucopyranoside, methyl-α-D-mannopyranoside, methyl- βD-xylopyranoside, amidone, amygdalin, arbutin, dulcitol, erythritol, esculin, gentiobiose, glycerol, glycogen, inositol, salicin, xylitol, L-arabinose, L-arabitol, L-fructose, L-rhamnose, L-sorbose, L-xylose, potassium gluconate, potassium 2-ketogluconate, potassium, and 5-ketogluconate.

The main fatty acid components identified from strain C17^T^ were hexadecanoic acid (C16:0, 24.31%), 9-octadecenoic acid (C18:1 n9, 13.25%), branched fatty acids (C18:1 n7/C18:1 n6, 13.16%), and octadecanoic acid (C18:0, 12.39%), which could also be detected in closely related *Streptococcus* species. 11-Hexadecenoic acid (C16:1 n5, 1.42%) was detected in the isolated C17^T^ strain rather than in other types of closely related species. A complete fatty acid analysis report of C17^T^ and other related species of the family *Streptococcaceae* are summarized in Table 3.

**Table 3:** Cellular fatty acid composition (%) of C17^T^ and other closely related species

Fatty acids	Name	1	2	3	4	5	6
C16:0	Hexadecanoic acid	24.31	35.5	31.45	36.54	32.34	34.2
C18:1 n9	9-Octadecenoic acid	13.25	11.35	12.75	10.59	11.05	14.86
Sum In Feature 8	18:1 n7/18:1 n6	13.16	7.43	10.02	6.97	6.51	5.96
C18:0	Octadecanoic acid	12.39	12.52	12.35	11.61	11.02	12.82
Sum In Feature 5	18:2 n6, 9/18:0 anteiso	8.92	6.29	7.41	5.46	7.77	7.35
Sum In Feature 3	16:1 n7/16:1 n6	8.54	3.12	1.55	3.15	3.57	2.89
C16:1 n9	7-Hexadecenoic acid	6.46	1.1	3.75	1.5	2.75	1
C14:0	Tetradecanoic acid	6.42	14.02	9.95	15.21	14.85	11.47
C12:0	Dodecanoic acid	1.48	4.95	TR	4.97	4.24	4.31
C16:1 n5	11-Hexadecenoic acid	1.42	TR	TR	TR	TR	TR
C20:4 n 6, 9, 12, 15	5, 8, 11, 14‐Eicosatetraenoic acid, etc.	1.19	TR	1.12	TR	1.19	TR
C17:0	Heptadecanoic acid	1.00	TR	TR	TR	1	1.05
C17:0 anteiso	14-Methyl-hexadecanoic acid	ND	TR	TR	TR	TR	TR
C20:1 n9	Cis-11-Eicosenoic acid	ND	ND	4.19	ND	ND	ND
C15:0	Pentadecanoic acid	TR	TR	1.34	TR	1.08	1.24
C15:0 anteiso	12-Methyl‐tetradecanoic acid	TR	ND	ND	TR	ND	ND
C17:0 iso	15-Methyl-Hexadecanoic acid	ND	ND	ND	TR	TR	ND
C13:0	Tridecanoic acid	ND	ND	ND	TR	TR	TR
C18:1 n5	13-Octadecenoic acid	ND	ND	TR	TR	ND	ND

Strains: 1, *S. symci* C17^T^; 2, *S. oralis* ATCC 35037^T^; 3, *S. infantis* ATCC 15192^T^; 4, *S.dentisani* DSM 27089^T^; 5, *S.australis* ATCC 700641^T^; 6, *S. pseudopneumoniae* ATCC BAA-960^T^. ND: not detected; TR: trace amounts < 1%.

In the antibiotic susceptibility test, strain C17^T^ was shown to be susceptible to clindamycin, linezolid, vancomycin, chloramphenicol, and cefepime, and the susceptibility of C17^T^ to erythromycin was determined to be moderate. C17^T^ was resistant to ceftriaxone, ampicillin, and cefotaxime.

### Genomic properties

**Fig. 4:**
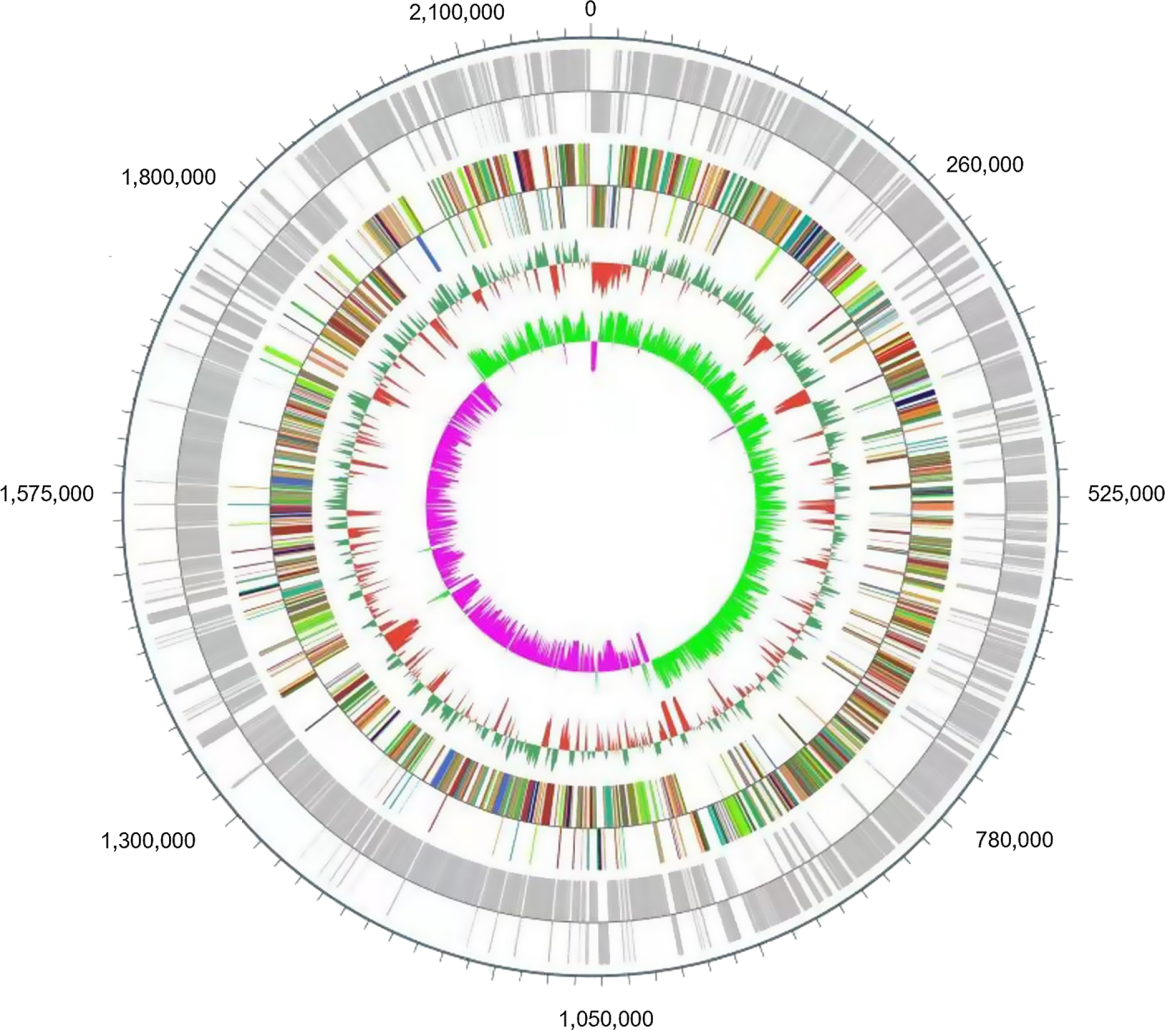
The genome graphical circular map of *S. symci* C17^T^. The outermost circle is the position coordinate of the genomic sequence. From outer to inner: coding DNA sequences on the forward strand (the outer chain), coding DNA sequences on the reverse strand (the inner chain), COG category of genes on the forward strand (the positive chain by the outer circle); COG category of genes on the reverse strand (the negative chain by the inner circle); genome GC content (inward red part indicates that the GC content in this area is lower than the whole genome average GC content, the outward green part is opposite), genomic GC skew value (pink part indicates that the area G content is lower than C Content, the outward light green part is opposite). (Color figure online)

The draft genome size of strain C17^T^ was 2,189,419 bp with a G+C content of 39.95% (Table 4, Fig. 4). It contained eight contigs covering 2092 predicted genes in total. Among these genes, 2057 were protein-coding genes, and 43 were genes coding for RNAs (including one 5S rRNA and 42 tRNA genes). A total of 340 genes were annotated as hypothetical proteins (16.53%). A total of 1782 genes (85.18%) were assigned to COGs, 201 of which were associated with virulence (9.61%). To search for potential secondary metabolite biosynthetic gene clusters (BGCs) in C17^T^, the genome sequence was unloaded to the antiSMASH program (version 2.0.2) for detailed screening. Only one BGC that was annotated as a bacteriocin was found in C17^T^. Meanwhile, eight CRISPR repeats were identified in the whole genome. Among the 25 general COG functional categories, five were not assigned to eight closely related species, including RNA processing and modification, chromatin structure and dynamics, nuclear structure, and cytoskeleton. Eight COG functional categories were grouped with more associated genes in C17^T^ than other closely related strains. The detailed distribution of genes was as follows: translation, 164 genes; amino acid transport and metabolism, 161 genes; cell wall/membrane biogenesis, 121 genes; nucleotide transport and metabolism, 73 genes; posttranslational modification, protein turnover, and chaperons, 71 genes; signal transduction mechanism, 56 genes; energy production and conversion, 52 genes. The genome statistics are presented in Table 4, and the gene distribution into COG functional categories is summarized in Table 5.

**Table 4:** Nucleotide content and gene counts of the genome of *S.symci* C17^T^

Attribute	Genome	
	Number	Total percentage (%)
Genome Size (bp)	2,189,419	100
G+C Content	874,673	39.95
Total number of genes	2092	100
Total number of protein‐coding genes	2057	98.33
Total number of RNA Genes	43	2.06
Total number of tRNA Genes	42	2.01
Total number of rRNA (5S, 16S, 23S) Genes	1	0.05
Coding sequence gene protein size (bp)	1981,482	90.50
Number of proteins associated with clusters of orthologous groups	1782	85.18
Number of proteins with peptide signal	88	4.21
Number of genes associated with virulence	201	9.61
Number of proteins with transmembrane helix	557	26.63
Genes associated to bacteriocin	12	0.57

**Table 5:** Number of genes associated with the 25 general clusters of orthologous group functional categories

Code	Description	*S. symci*	S. *mitis*	*S. oralis*	*S. dentisani*	*S. pneumoniae*	*S. pseudopne* *umoniae*	*S. infantis*	*S. tigurinus*
J	Translation	164	146	147	146	0	0	0	0
A	RNA processing and modification	0	0	0	0	0	0	0	0
K	Transcription	111	116	101	106	I28	129	91	126
L	Replication, recombination and repair	117	111	94	115	199	153	112	157
B	Chromatin structure and dynamic	0	0	0	0	0	0	0	0
D	Cell cycle control, mitosis and meiosis	21	19	23	20	21	21	23	21
Y	Nuclear structure	0	0	0	0	0	0	0	0
V	Defense mechanisms	70	46	40	53	72	81	47	58
T	Signal transduction mechanism	56	52	45	50	46	55	46	49
M	Cell wall/membrane biogenesis	121	95	95	91	118	89	105	101
N	Cell motility	3	1	3	2	1	1	2	2
Z	Cytoskeleton	1	0	0	0	0	0	0	1
w	Extracellular structures	1	II	n	II	II	II	II	1
U	Intracellular trafficking and secretion	27	20	30	25	25	22	22	37
O	Post translational modification, protein turnover, chaperones	71	59	62	53	65	62	52	59
C	Energy production and conversion	52	48	41	41	49	41	40	46
G	Carbohydrate transport and	129	130	112	125	194	152	111	140
E	Amino acid transport and metabolism	161	137	145	155	154	147	I28	142
F	Nucleotide transport and metabolism	73	65	68	68	65	67	65	70
H	Coenzyme transport and metabolism	48	41	41	43	43	50	36	43
I	Lipid transport and metabolism	32	30	30	31	31	30	30	33
P	Inorganic ion transport and	100	93	90	88	114	101	76	94
Q	Secondary metabolites biosynthesis, transport and Catabolism	13	10	11	11	12	12	11	15
R	General function prediction only	0	0	0	0	0	0	0	0
S	Function unknown	412	339	384	377	384	406	380	440

### Genomic comparative analysis between C17^T^ and closely related species

**Fig. 5:**
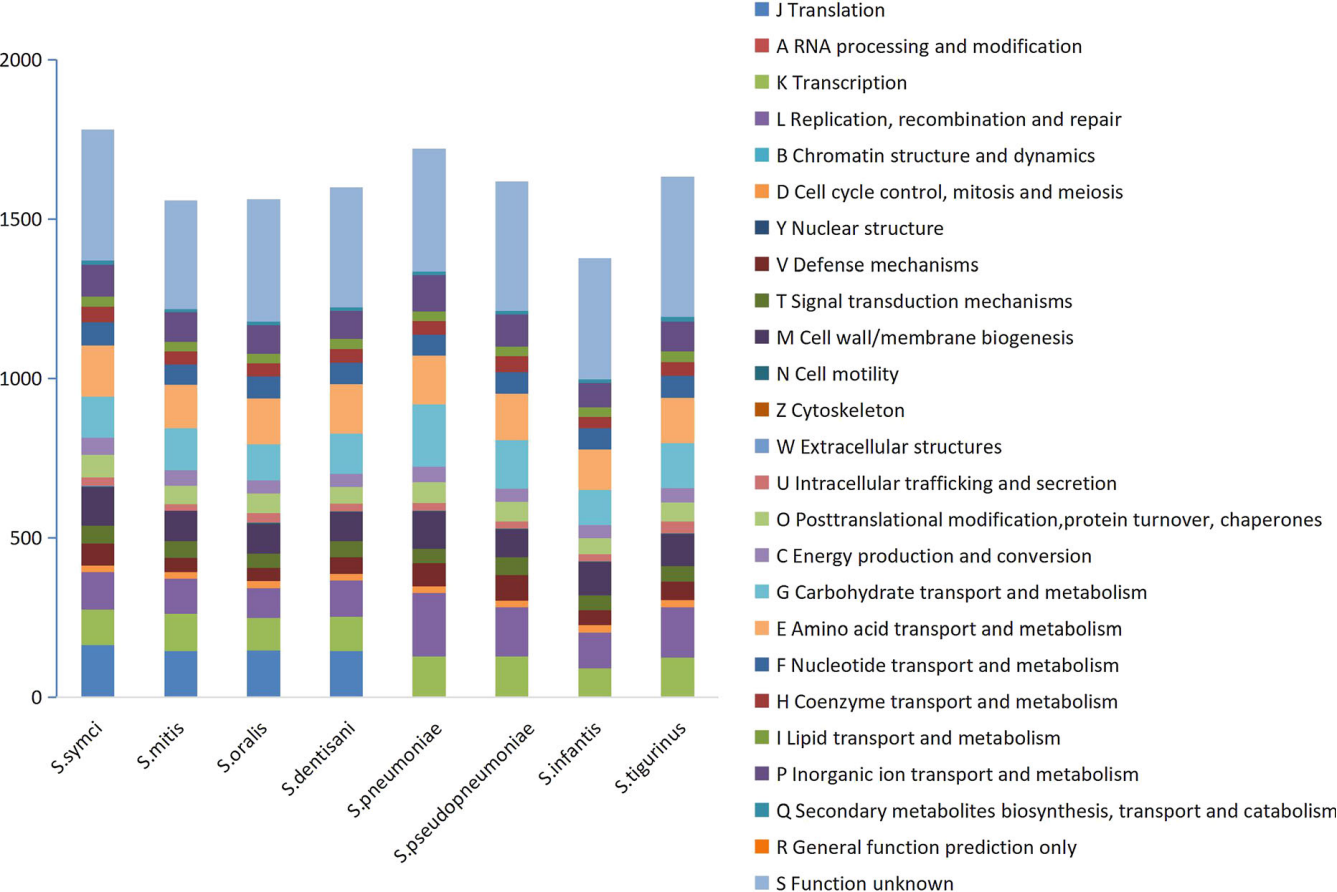
Distribution of the predicted genes of *S. symci* C17^T^ to different COG functional categories, compared to other closely related species

To calculate the dDDH between C17^T^ and other available species that are phylogenetically closest (Table 6), the GGDC online formula 2 calculator was used for detailed comparative analysis. Strain C17^T^ displayed dDDH values of 47.20, 52.90, 30.90, 44.90, and 26.20 for *S. pseudopneumoniae* ATCC BAA-960^T^, *S. mitis* ATCC 49456^T^, *S. oralis* ATCC 35037^T^, *S. pneumoniae* NCTC 7465^T^, and *S. infantis* ATCC 15192^T^, respectively. These dDDH values were lower than the threshold value of 70% for species demarcation. The pair-wise ANI values between strain C17^T^ and the type strain of other *Streptococcus* species were 91.99%, 93.40%, 85.90%, 91.42% and 82.21% respectively, thereby indicating that the newly isolated strain is representative of a new *Streptococcus* species. The distribution of the predicted genes of *S. symci* C17^T^ to different COG functional categories is summarized in Fig. 5.

**Table 6:** The dDDH values (%) obtained by a comparative analysis of *Streptococcus symci* C17^T^ and other closely related species (calculated by GGDC formula 2, DDH was estimated based on identity/HSP length)

	*S.symci*	*S. pseudopneumoniae* (%)	*S. mitis* (%)	*S. oralis* (%)	*S. pneumonia* (%)	*S. infantis* (%)
*S. symci*	100	47.20 (44.6–49.8)	52.90 (50.3–55.6)	30.90 (28.5–33.4)	44.90 (42.4–47.5)	26.20 (23.9–28.7)
*S. pseudopneumoniae*		100	48.20 (45.6–50.8)	31.80 (29.4–34.3)	58.80 (56–61.6)	26.40 (24.1–28.9)
*S. mitis*			100	31.70 (29.3–34.2)	46.30 (43.7–48.8)	26.10 (23.7–28.6)
*S. oralis*				100	31.50(29.1–34.1)	29.60(26.7–32.7)
*S. pneumoniae*					100	25.90(23.5–28.3)
*S. infantis*						100

## Discussion

Recognized as an important part of commensal microbiota in humans, *Streptococcus* species are widely distributed in all parts of the human body, especially the mouth, skin, intestine, and upper respiratory tract. They are responsible for many types of diseases, including meningitis, pneumonia, and erysipelas (Krzysciak et al. 2013). However, many *Streptococcus* species are nonpathogenic symbionts. Here, we isolated a *Streptococcus* strain C17^T^ from the oropharynx mucosa sample of a healthy 5-year-old child. Phenotypic and biochemical feature identification, phylogenetic analysis, and genome annotation were performed. The results indicated that C17^T^ was a new species of the *Streptococcus* genus.

Set as a key criterion, a 70% threshold of dDDH value has been adopted to delimitate a species (Auch et al. 2010; Meier-Kolthoff et al. 2013; Wayne 1988). The dDDH values of C17^T^ with other adjacent strains calculated by GGDC (online formula 2 calculator) were all less than 70%. Among all the comparative analyses, the dDDH for estimating the genomic distance between strain C17^T^ and the nearest *S. pseudopneumoniae* (16S rRNA closest species with standing in nomenclature) was 47.2%, while the value for estimating C17^T^ compared with *S.mitis* (Gro EL, rpoB, and sodA genes) was 52.9%. For the comparative analysis of C17^T^ with other *Streptococcus* species, the dDDH values were even lower, 30.9% for *S. oralis* ATCC35037^T^, 44.90% for *S. pneumoniae* NCTC7465^T^, and 26.20 for *S. infantis* ATCC15192^T^ (Table 6).The genome sequence of strain C17^T^ had 82.21–93.40% ANI with type strains of other S*treptococcus* species (Table 7), which are below the ≥ 95% ANI cut-off to define a bacterial species (Richter and Rossello-Mora 2009). Thus, the results of genome distance analysis provide strong evidence supporting the identification of *S. symci* C17^T^ as a new *Streptococcus* species. The results of phenotype analysis obtained through API strips indicated that strain C17^T^ possessed unique profiles of enzyme spectra and sugar utilization for fermentation, compared with other closely related species (Table 2). The biochemical features of other neighboring strains were consistent with those reported in the literature (Huch et al. 2013). The fatty acid composition of C17^T^ was also clearly distinct from other closely related species, indicating the unique metabolome profile of C17^T^. 16S rRNA can only be used for strain identification for classification up to the genus level; thus, among all *Streptococcus* species, the similarity of C17^T^ to other *Streptococcus* species with highly homologous 16S rRNA was a common feature (Fig. 1). Meanwhile, the gene comparison analysis of concatenated *groEL*, *rpoB*, and *sodA* demonstrated high sequence identity with the closest *S.mitis* strain ATCC 49456^T^ (Fig. 2). This result was consistent with that of the DDH analysis. The genomic analysis of C17^T^ showed that eight COG functional categories were distributed with more associated genes in C17^T^, compared with the other closely related species, indicating that C17^T^ is different from the other known *Streptococcus* species at the genetic level.

**Table 7:** ANI values (%) between *S. symci* C17^T^ and other closely related species (calculated by the OrthoANI algorithm)

	*S. symci*	*S. pseudopneumoniae* (%)	*S. mitis* (%)	*S. oralis* (%)	*S. pneumonia* (%)	*S. infantis* (%)
*S. symci*		91.6712	93.1873	85.3982	90.8849	81.5349
*S. pseudopneumoniae*			81.4333	81.3882	81.2183	81.6869
S. mitis				85.76	91.4778	92.1375
*S. oralis*					85.4564	85.6727
*S. pneumoniae*						94.44
*S. infantis*						

In this study, the preliminary tests showed that strain C17^T^ had the effect of antagonizing pathogens, which was different from that of *S. mitis*. Together with other studies on strain characteristics, it was concluded that the classification of strain C17^T^ was similar, but still did not belong to *S. mitis.* Unfortunately, due to the high genomic similarity with various pathogenic *Streptococcus* genera, we still have no adequate evidence to rule out the possibility that this bacterium is virulent, and no virulence factor analysis was conducted in this study. However, strain C17^T^ is an independent species in the *Streptococcus* genus and may develop into a type of probiotic that is necessary for maintaining the health of the human oropharynx.

## Description of *S. symci* sp. nov.

*Streptococcus symci* (sym'ci. N.L. gen. n. symci, arbitrary epithet derived from Shenyang Medical College, where the sample was characterized).

It is a non-motile, non-spore-forming, aerobic, and Gram-positive bacteria with an approximate diameter of 5 μm. The cells formed grass-green, α-hemolytic colonies on Columbia agar plates containing 5% sheep blood after 24 h of incubation. The cells can grow at a temperature ranging from 20 to 42 °C with an optimal temperature of 37 °C under anaerobic, microaerophilic, and aerobic conditions. The growth of C17^T^ was observed at different pH values (from 5.0 to 8.5) and salt concentrations of up to 2.5% NaCl. No oxidase and catalase activities were detected. The major fatty acids were hexadecanoic acid (24.31%), 9-octadecenoic acid (13.25%), branched fatty acids C18:1 n7/C18:1 n6 (13.16%), and octadecanoic acid (12.39%).

This strain of *Streptococcus symci* sp. nov., C17^T^, was first isolated from the oropharynx of a healthy 5-year-old child in Shenyang. The size of the genome was 2,189,419 bp, with a DNA G+C content of 39.95%. The 16S rRNA sequences of C17^T^ were uploaded to the GenBank with the accession number MN068913.1. The whole-genome shotgun sequence was uploaded to the GenBank under accession number VFJA00000000. The habitat of this bacterium was a healthy oropharynx.
